# Which biomarker predicts benefit from EGFR-TKI treatment for patients with lung cancer?

**DOI:** 10.1038/sj.bjc.6603665

**Published:** 2007-02-27

**Authors:** H Uramoto, T Mitsudomi

**Affiliations:** 1 Cancer Chemotherapy Center, University of Occupational and Environmental Health, Japan. Yahatanishi-ku Kitakyushu 807-8555, Japan; 2 Department of Thoracic Surgery, Aichi Cancer Center Hospital, 1-1, Kanokoden, Chikusa-ku, Nagoya 464-8681, Japan

**Keywords:** gefitinib, erlotinib, biomarker, clinical trials, individualized therapy

## Abstract

Subsets of patients with non-small cell lung cancer respond remarkably well to small molecule tyrosine kinase inhibitors (TKI) specific for epidermal growth factor receptor (EGFR) such as gefitinib or erlotinib. In 2004, it was found that *EGFR* mutations occurring in the kinase domain are strongly associated with EGFR-TKI sensitivity. However, subsequent studies revealed that this relationship was not perfect and various predictive markers have been reported. These include *EGFR* gene copy numbers, status of ligands for EGFR, changes in other *HER* family genes or molecules downstream to EGFR including KRAS or AKT. In this review, we would like to review current knowledge of predictive factors for EGFR-TKI. As all but one phase III trials failed to show a survival advantage of the treatment arm involving EGFR-TKIs, it is necessary to select patients by these biomarkers in future clinical trials. Through these efforts, it would be possible to individualise EGFR-TKI treatment for patients suffering from lung cancer.

Lung cancer is the leading cause of cancer-related mortality in Japan as well as in Western countries. The high mortality is mainly because of early development of systemic disease and resistance to currently available treatment strategies. Although various chemotherapeutic agents were developed in the late 1980s and 1990s, platinum doublet therapy seems to reach a therapeutic plateau with an objective response rate of 30–40% and a median survival time (MST) of 8–10 months for patients with stage IIIB or IV disease.

To circumvent this situation, a new class of drugs that specifically targets certain molecular pathways leading to cancer phenotypes is being actively developed. The epidermal growth factor receptor (EGFR) pathways have been investigated as a potential target for cancer therapy because EGFR overexpression is frequently observed and associated with a poor prognosis or resistance to chemotherapy. Antibodies directed against the extracellular domain of EGFR (such as cetuximab, matuzumab and panitumab) and small-molecule tyrosine kinase inhibitors (TKIs) that target the kinase domain (such as gefitinib and erlotinib) are in clinical use or in a late developmental stage.

In the phase II trail of gefitinib and early clinical development, subgroups of patients who are of Asian origin, female sex, adenocarcinoma and no history of smoking have been significantly associated with a favourable response to TKIs (Fukuoka *et al*, 2003; Kris *et al*, 2003; Miller *et al*, 2004). An analysis of 1974 patients taken from the literature (Mitsudomi *et al*, 2006) indicated that the TKI response is significantly dependent on ethnicity (Caucasian 10% *vs* East Asians 33%), gender (male 13% *vs* female 33%), smoking history (never smoker 40% *vs* current/former smokers 11%), and histologic type (adenocarcinoma 29% *vs* nonadenocarcinoma 5%). However, it was not possible to predict gefitinib sensitivity by levels of EGFR overexpression, determined by immunohistochemistry or immunoblotting. The factors that determine gefitinib sensitivity have long been an enigma.

## *EGFR* MUTATIONS

In 2004, it was found that a subset of pulmonary adenocarcinoma has somatic, activating mutations of the *EGFR* gene (Lynch *et al*, 2004; Paez *et al*, 2004; Pao *et al*, 2004). Following these initial reports, various groups confirmed and extended the findings that *EGFR* mutations are found in the first four exons of the tyrosine kinase (TK) domain of the *EGFR* gene and about 90% of these *EGFR* mutations are either short, in-frame deletions in exon 19 or point mutations that result in a substitution of arginine for leucine at amino acid 858 (L858R). *EGFR* mutations were predominantly found in female subjects, nonsmokers, adenocarcinomas, and Japanese patients (for review, see Mitsudomi *et al*, 2006). These patient characteristics precisely coincide with those with a high response rate to EGFR-TKIs described above. Of particular interest, *EGFR* mutation is the first molecular abnormality that is more frequent in nonsmoking patients with non-small cell lung cancer (NSCLC). However, this does not necessarily mean that smoking has a protective effect for *EGFR* mutations. Our case–control study revealed that lung cancers harbouring *EGFR* mutation appear to occur independent of tobacco smoking, whereas lung cancers without *EGFR* mutations are very much dependent on smoking dose (Matsuo *et al*, 2007). Apparent inverse relationship between smoking and *EGFR* mutations was thus due to dilutional effect of *EGFR*-mutated tumours by *EGFR* nonmutated tumours (Matsuo *et al*, 2007).

When *EGFR* mutations were first reported, the most exciting finding was that lung cancer harbouring this genetic alteration showed a striking response to EGFR-TKIs (Lynch *et al*, 2004; Paez *et al*, 2004; Pao *et al*, 2004). According to the data for 1170 patients, more than 70% of NSCLCs with *EGFR* mutations respond to EGFR-TKIs, whereas 10% of tumours without *EGFR* mutations do so (Table 1). Furthermore, several investigators have reported that patients with *EGFR* mutations have a significantly longer survival than those with wild-type EGFR when treated with EGFR-TKIs (Table 1). However, data on predictors for survival are controversial. Some investigators claim that EGFR mutations are prognostic rather than predictive, because subset analysis of TRIBUTE or INTACT trials (comparing platinum chemotherapy with chemotherapy plus EGFR-TKI) indicated that patients with lung cancer having *EGFR* mutations did better even in patients treated only with chemotherapy (Bell *et al*, 2005; Eberhard *et al*, 2005). However, *EGFR* mutations was not a significant prognostic factor in an initial two large retrospective studies in surgically treated patients without gefitinib treatment (Kosaka *et al*, 2004; Shigematsu *et al*, 2005), although Shigematsu *et al* (2005) reported that patients with exon 19 deletion have significantly shorter survival than those with L858R, but this is not confirmed by other investigators so far. These results clearly show that *EGFR* mutations are important in determining EGFR-TKI sensitivity, although not perfect. High response rate in patients with *EGFR* mutations to gefitinib was confirmed in the recently published prospective phase II study (Inoue *et al*, 2006 and Table 1).

We first reported that response rate of gefitinib is higher for patients with deletional *EGFR* mutations than for those with other types of mutations, predominantly L858R (Mitsudomi *et al*, 2005) and others extended this observation by demonstrating survival difference between them (Jackman *et al*, 2006; Riely *et al*, 2006). On the other hand, one of the insertion mutation (D770insNPG) in exon 20 of the *EGFR* gene has been shown to be associated with *in vitro* resistance to erlotinib (Greulich *et al*, 2005). In this study, G719S of exon 18 showed intermediate sensitivity, suggesting the mutation-specific treatment strategy for patient care.

Two groups of researchers have recently developed transgenic mice that express either exon 19 deletion mutant or the L858R mutant in type II pneumocytes under the control of doxycycline (Ji *et al*, 2006; Politi *et al*, 2006). Expression of either *EGFR* mutant leads to the development of adenocarcinoma similar to human bronchioloalveolar cell carcinoma and withdrawal of doxycycline to reduce expression of transgene or erlotinib treatment resulted in tumour regression. Thus, these experiments showed that persistent EGFR signalling is required for tumour maintenance in human lung adenocarcinomas expressing *EGFR* mutants.

## *EGFR* GENE COPY NUMBER

Cappuzzo *et al* (2004) reported that increase in *EGFR* gene copy number, as determined by fluorescence *in situ* hybridisation, is more predictive of the patient survival after gefitinib treatment than *EGFR* mutations (Cappuzzo *et al*, 2005a). However, this report does not necessarily refute the role of *EGFR* mutations as a predictive factor because *EGFR* mutations only failed to significantly affect overall survival (*P*=0.09), whereas EGFR mutations were predictive of response rate and time to progression (Cappuzzo *et al*, 2005a). However, it should be noted that their definition of increased gene copy number included both gene amplification and high polysomy (more than 40% of tumour cells have more than four copies of the EGFR gene). It is biologically unclear whether high polysomy indicates the activation of the EGFR gene, resulting in effects similar to those caused by gene amplification. Tsao *et al* (2005) reported that increased *EGFR* gene copy number is most predictive of a longer survival in patients who received erlotinib in a phase III clinical trial (BR.21) that compared erlotinib with best supportive care. They concluded that the detection of *EGFR* mutations is not necessary in selecting patients who will benefit from erlotinib therapy (Tsao *et al*, 2005). Recently it was reported that *EGFR* gene copy number but not gene mutation was the predictor of clinical benefit from gefitinib in ISEL, a similar randomised trial comparing gefitinib with placebo (Hirsch *et al*, 2006).

However, many investigators refute this point. Han *et al* (2006) recently reported that *EGFR* mutation and high gene copy number were associated with better objective response in univariate analysis. Only gefitinib-sensitive *EGFR* mutation was independently predictive of both response and survival in multivariate analysis. Furthermore, Tsao *et al* (2005) report that 53% of the *EGFR* mutations they found were novel variant mutations, of which 92% were C/G → T/A or A/T → G/C transitions. Marchetti *et al* (2006) suggested that at least some of these mutations could be artifactual if Tsao *et al* (2005) used small amount of DNA from paraffin-embedded tissues. Tsao *et al*, (2005) responded to this comment by stating that even when the mutation analysis was confined to patients with exon 19 deletion and L858R, overall results did not change, confirming no association of response and survival for mutations in the BR.21 trial (Tsao *et al*, 2005). In general, tumours with *EGFR* mutations tend to have gene amplification. Mutation and amplification are probably both important in determining TKI sensitivity. To resolve this controversy, both *EGFR* mutations and amplification should be determined prospectively in future clinical trials. These results are also summarised in Table 1.

## OTHER MOLECULAR PARAMETERS

### Ligands

Using cDNA microarray, increased expression of amphiregulin or TGF-*α*, known to be the ligands for EGFR, is related with poor response to gefitinib (Kakiuchi *et al*, 2004). Recently, it was reported that heregulin, ligand for HER3, expression correlates with gefitinib insensitivity (Zhou *et al*, 2006). The ADAMs (a disintegrin and metalloproteases), zinc-dependent membrane-associated proteases, control the cleavage of most EGF-related ligands. Zhou *et al* (2006) also showed that ADAM17 protein is upregulated in NSCLC and correlated with heregulin-mediated HER3 activation, leading to gefitinib insensitivity

### Receptors

In addition to *EGFR* gene copy numbers, Cappuzzo *et al* (2005c) reported that increased *HER2* gene copy number is associated with response to gefitinib therapy in *EGFR*-mutated NSCLC. However, the same group reported that genomic gain for *HER3* is not a marker for response or resistance to TKI therapy in advanced NSCLC patients (Cappuzzo *et al*, 2005b). It is also reported that cancer cells having *HER2* mutations, present in a very small fraction of NSCLC, are insensitive to EGFR-TKI, but remain sensitive to *HER2*-targeted therapies (Wang *et al*, 2006).

### Downstream molecules

Pao *et al* (2005b) first reported that lung cancers with *KRAS* mutations are resistant to EGFR-TKIs. None of the nine tumours with *KRAS* mutations responded to EGFR-TKIs (Pao *et al*, 2005b). However, Miller *et al* (2006) showed that in their study of adenocarcinoma with bronchioloalveolar cell feature treated with erlotinib, some of the tumours with *KRAS* mutations showed minor tumour shrinkage, although response rate of lung cancer with *KRAS* mutations was zero by RECIST. Thus, it is not possible to exclude patients with *KRAS* mutations from the list of patients with potential clinical benefit from EGFR-TKI therapy.

AKT is phosphorylated on EGFR activation, transmitting signals for cell survival. It is reported that patients with phospho AKT-positive tumours had a better response rate, disease control rate, and time to progression by gefitinib treatment (Cappuzzo *et al*, 2004). Expression of E-cadherin, a calcium-dependent adhesion molecule, has been related to sensitivity to gefitinib (Witta *et al*, 2006). These lines of evidence clearly indicate that comprehensive analyses of molecular biomarkers should be carried out in conjunction with clinical trials of EGFR inhibitors.

### RESISTANCE TO EGFR-TKI

In contrast to the inherent resistance to gefitinib such as that by *KRAS* mutations described above, it is common for patients to show progressive disease after presenting with an initial good response. A secondary mutation resulting in threonine to methionine at codon 790 (T790M) has been reported to be responsible for the acquired resistance (Kobayashi *et al*, 2005; Pao *et al*, 2005a). Crystal structure modelling has revealed that position T790 is located in the ATP-binding pocket of the catalytic region and appears to be critical for the binding of erlotinib and gefitinib. Substitution of the threonine at this codon with a bulkier residue, such as methionine, is thought to sterically hinder the binding of these two drugs. We recently reported that out of 14 patients who developed acquired resistance after initial good response, seven patients harboured additional T790M mutation (Kosaka *et al*, 2006). However, we were not able to find novel mutations related to this resistance, which is in good contrast with resistance in imatinib treatment for chronic myeloid leukaemia where more than 30 mutations of the *ABL* gene have been reported. In tumours from patients not treated with TKI, T790M appears to be rare, approximately 0.5% (Toyooka *et al*, 2005). The possibility exists, however, that this second mutation might be present at a low frequency at the time of diagnosis and that tumour cells harbouring this mutation might be enriched over time during treatment with gefitinib (Inukai *et al*, 2006).

### TKIs AND CLINICAL TRIALS

The addition of TKIs (gefitinib or erlotinib) did not yield a survival advantage over platinum doublet (carboplatin/paclitaxel or cisplatin/gemcitabine) in four randomised trials (INTACT I, II TALENT, and TRIBUTE). However, subgroup analysis of the TRIBUTE trial showed that the addition of erlotinib to carboplatin plus paclitaxel conferred an advantage in overall survival in patients who were never-smokers (MST 22.5 months *vs* 10.1 months; *P*=0.01) (Herbst *et al*, 2005).

In a randomised placebo-controlled trial to determine whether erlotinib prolonged survival in patients with NSCLC after the failure of chemotherapy (BR.21), erlotinib significantly prolonged survival, with an MST of 6.7 months *vs* 4.7 months (hazard ratio 0.70; *P*<0.001) (Shepherd *et al*, 2005). In contrast, a similar placebo-controlled randomised trial using gefitinib (ISEL trial) failed to show an overall survival advantage in the gefitinib treatment group (MST of 5.6 months *vs* 5.1 months; *P*=0.087) (Thatcher *et al*, 2005). However, gefitinib prolonged survival in never-smokers (MST 8.9 months *vs* 6.1 months; *P*=0.012) as well as in Asian patients (MST 9.5 months *vs* 5.5 months; *P*=0.010) in preplanned subset analyses (Thatcher *et al*, 2005). Following these results, the US Food and Drug Administration limits the indication of gefitinib to cancer patients who are currently benefiting or have previously benefited from gefitinib treatment or are enrolled in clinical trials as of June 2005.

As has been described, EGFR-TKIs are not universally effective for lung cancer, but these drugs are effective in patients who have particular clinical or biological characteristics, for example, Asian, nonsmoking female patients with adenocarcinomas with *EGFR* mutations. The different outcomes of the BR.21 and ISEL trials are at least partly attributable to differences in the degree of dilution in the two trials of patients with the above-mentioned characteristics by those without such characteristics. Therefore, patients who would benefit from EGFR-TKI therapy should be concentrated in future clinical trials. Smoking history and *EGFR* mutations are good predictors of response in patients treated with EGFR-TKIs. Which of these two markers should we use in future clinical trials? In our exploratory subset analysis, tumour response was observed in 16 out of 19 patients with both *EGFR* mutations and no smoking history (Mitsudomi *et al*, 2005). Whereas a response was seen in one out of six never-smokers without *EGFR* mutations, a response was seen in eight out of 10 smokers with *EGFR* mutations (Mitsudomi *et al*, 2005). Therefore, our limited experience indicates that *EGFR* mutations may be superior to smoking history in the selection of patients who would benefit from TKI treatment and that smoking history is only a surrogate marker of *EGFR* mutation. Obviously, the detection of *EGFR* mutations requires laborious laboratory work. Hence, smoking history can be used in contexts in which *EGFR* gene testing is not readily available. In April 2004, IPASS (iressa pan-Asian study) was started. This is an open-labelled randomised phase III study comparing gefitinib monotherapy with cariboplatin/paclitaxel for previously untreated patients with adenocaricnoma who are never- or light smokers. The West Japan Thoracic Oncology Group, launched a phase III clinical trial comparing gefitinib monotherapy with cisplatin plus docetaxel in lung-cancer patients with *EGFR*. Primary end point is progression-free survival, to avoid confounding by possible crossover between two arms and the sample size is 200 patients with *EGFR* mutations. We also limit our mutation search to deletions in exon 19 and L858R, because it would be less laborious and these two are most reliable predictor for response or survival. In this way, the survival benefit of EGFR-TKIs, especially gefitinib, should be demonstrated in future clinical trials in a defined subset of patients with lung cancer.

## LIFE-THREATENING INTERSTITIAL LUNG DISEASE

In Japan, soon after the introduction of gefitinib, life-threatening interstitial lung disease or acute lung injury attributable to gefitinib became apparent. Recently published retrospective survey of 1976 Japanese patients showed prevalence and morality of ILD was 3.5 and 1.6%, respectively (Ando *et al*, 2006). Gefitinib-induced ILD was significantly associated with male sex, a history of smoking, and coincidence of interstitial pneumonitis (odds ratios 3.10, 4.79, and 2.89, respectively) (Ando *et al*, 2006). Although it is not very clear whether the high incidence of ILD is common in East Asian countries, Chiu *et al* (2005) reported from Taiwan that geftinib-related interstitial pneumonia was clinically diagnosed in four cases (5.8%) in 69 patients. Biomarkers including genetic polymorphisms that predict occurrence of ILD should be actively sought. The incidence of ILD by erlotinib appears similar in Japan. In a recent phase II trial of erlotinib conducted in Japan, possible ILD-like events were reported in four of 60 evaluable patients (6.7%)(Tamura *et al*, 2006), whereas ILD was reported in three of 485 in BR. 21 (Shepherd *et al*, 2004).

## CONCLUSIONS

**Figure 1:**
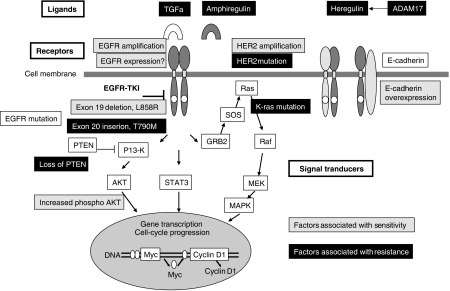
Potential molecular biomarkers to predict responsiveness for EGFR-TKI in EGFR signalling pathways. Sensitive and resistant markers are indicated by grey and black boxes, respectively.

Considering great complexity and redundancy of EGFR pathway, it is natural to assume that one cannot expect a sole determinant of clinical benefit of EGFR-TKIs. Figure 1 summarises current knowledge of molecular predictors for EGFR-TKI discussed above. The development of EGFR-TKIs and the discovery of *EGFR* gene mutations have provided a great opportunity for translation of cancer biology into clinics to realise individualised therapies for lung cancer. However, we should continue our effort to search for better biomarker(s) that is most clinically relevant. Particularly in Japan, risk of ILD and potential benefit of EGFR-TKI therapy should be well balanced.

## Figures and Tables

**Table 1:** Effect of mutations and copy number of the EGFR gene on clinical outcome in patients treated with EGFR-TKIs

				**Mutational status**												**Copy number**													
				**Tumour response**						**TTP**			**OS**					**Tumour response**						**TTP**			**OS**		
				**Mut**		**Wt**		**RR**										**High**		**Low**		**RR**							
**Investigator**	**Source**	**TKI**	**N**	**R**	**NR**	**R**	**NR**	**Mut**	**Wt**	**Mut**	**Wt**	**P**	**Mut**	**Wt**	**P**	**N**	**Method**	**R**	**NR**	**R**	**NR**	**High**	**Low**	**High**	**Low**	**P**	**High**	**Low**	**P**
*Retrospective analyses of patients from a single institution*																													
Paez	*Science* 2004	G	9	5	0	0	4	100	0																				
Lynch	*NEJM* 2004	G	16	8	0	1	7	100	13																				
Pao	*PNAS* 2004	G	18	7	0	3	8	100	27																				
Pao	*PNAS* 2004	E	17	5	0	2	10	100	17																				
Huang	*JCO* 2004	G	16	7	1	2	6	88	25																				
Tokumo	*CCR* 2005	G	21	8	1	2	10	89	17				25.1	14	0.15														
Mitsudomi	*JCO* 2005	G	50	24	5	2	19	83	10				NR	14	0.0053														
Han	*JCO* 2005	G	90	11	6	10	63	65	14	21.7	1.7	<0.001	30.5	6.6	<0.001														
Kim	*CCR* 2005	G	27	6	0	2	19	100	10				47.3	11.9	0.008														
Cortes-Funes	*Ann Oncol* 2005	G	78	6	4	6	62	60	9				13	4.9	0.02														
Cappuzzo	*NEJM* 2005	G	89	8	7	4	70	53	5	9.9	2.7	0	20.8	8.5	0.09	102	FISH	12	21	2	67	36	3	9.0	2.5	<0.001	18.7	7.1	0.03
Chou	*CCR* 2005	G	54	17	16	4	17	52	19				14.5^a^	4^a^	0.046														
Taron	*CCR* 2005	G	65	16	1	6	42	94	13				NR	9.9	0.001														
Takano	*JCO* 2005	G	66	32	7	3	24	82	11	12.6	1.7	<0.0001	20.4	6.9	0.0001	66	qPCR	21	8	14	23	72	38	9.4	26.0	0.038			0.49
Zhang	*Ann Oncol* 2005	G	30	8	4	1	17	67	6				NR	7	0.0022														
Mu	*CCR* 2005	G	22	7	3	0	12	70	0																				
Tomizawa	*CCR* 2005	G	22	12	0	4	6	100	40																				
Han	*CCR* 2006	G														66	qPCR	10	21	4	31	32	11	3.6	1.9	0.21	12.3	8.4	0.49
																													
*Retrospective analyses of patients enrolled in multiinstitutional clinical trials*																													
Bell	*JCO* 2005	G	80	6	7	6	61	46	9	5.5^a^	1.9^a^	S	7.9^a^	6.1^a^	NS	86	qPCR	2	5	12	67	29	15	5.5^a^	2.0^a^	S	8.1^a^	6.2^a^	NS
Tsao	*NEJM* 2005	E	100	3	16	6	75	16	7				7.5^a^	8.8^a^	NS	66	FISH	5	20	1	40	20	2				10.7^a^	7.8^a^	S
Hirsch	*JCO* 2006	G	132	6	10	3	113	38	3	Insufficient data for survival analysis						222	FISH	11	56	5	150	16	3	4.5	2.4		8.3	4.3	S
Hirsch	*JCO* 2005	G														55	FISH	5	14	4	32	26	11	9.0	4.0	0.072	NR	8.0	0.042
																													
*Prospective study for EGFR mutation as a predictor of tumour response*																													
Paz-Ares	*ASCO* 2006	E	38	31	7			82																					
Okamoto	*ASCO* 2006	G	27	20	7			74																					
Sutani	*ASCO* 2006	G	35	21	6	1	7	78	13																				
Morikawa	*ASCO* 2006	G	47	21	13	2	11	62	15																				
Yoshida	*JTO* 2007	G	21	19	2			90																					
																													
Totals			1170	314	123	70	663	72	10							663		66	145	42	410	31	9						

Abbreviations: E, erlotinib; EGFR, epidermal growth factor receptor; FISH, fluorescent *in situ* hybridisation; G, gefitinib; Mut, mutation; N, number of patients; NR, non-responder; NS, not significant; OS, Overall survival; qPCR, quantitative PCR; RR, response rate; TKI, tyrosine kinase inhibitor; TTP, time to progression; WT, wild-type; R, responder; S, significant.

a Read directly from graphs.
